# Patterns of risk for anxiety-depression amongst Vietnamese-immigrants: a comparison with source and host populations

**DOI:** 10.1186/1471-244X-13-329

**Published:** 2013-12-02

**Authors:** Belinda J Liddell, Tien Chey, Derrick Silove, Thuy Thi Bich Phan, Nguyen Mong Giao, Zachary Steel

**Affiliations:** 1 Psychiatry Research and Teaching Unit (PRTU), School of Psychiatry, University of New South Wales, Sydney, Australia; 2 School of Psychology, University of New South Wales, Sydney, Australia; 3 Centre for Population Mental Health Research, Level 1, Mental Health Centre, Liverpool Hospital, Sydney, Australia; 4 Department of Psychiatry, Cần Thơ University, Cần Thơ, Vietnam

**Keywords:** Anxiety, Depression, Risk factor, Immigrant, Low-middle income countries (LMIC), High income countries (HIC), Age, Trauma, Culture, Vietnam

## Abstract

**Background:**

Studies suggest that immigrants have higher rates of anxiety-depression than compatriots in low-middle income countries and lower rates than populations in host high income countries. Elucidating the factors that underlie these stepwise variations in prevalence may throw new light on the pathogenesis of anxiety-depressive disorders globally. This study aimed to examine whether quantitative differences in exposure to, or the interaction between, risk factors account for these anxiety-depression prevalence differences amongst immigrant relative to source and host country populations.

**Methods:**

Multistage population mental health surveys were conducted in three groups: 1) a Vietnamese-immigrant sample settled in Australia (n = 1161); 2) a Vietnamese source country sample residing in the Mekong Delta region (n = 3039); 3) an Australian-born host country sample (n = 7964). Multivariable logistic regression analyses compared risk factors between the Vietnamese-immigrant group and: 1) the Mekong Delta Vietnamese; and 2) the Australian-born group. Twelve month anxiety-depression diagnoses were the main outcome measures, derived from the Composite International Diagnostic Interview (CIDI), supplemented by an indigenously derived measure - the Phan Vietnamese Psychiatric Scale (PVPS) in both Vietnamese groups.

**Results:**

The 12-month prevalence of anxiety-depression showed a stepwise increase across groups: Mekong Delta Vietnamese 4.8%; Vietnamese-immigrants 7.0%; Australian-born 10.2%. The two Vietnamese populations showed a similar risk profile with older age, exposure to potentially traumatic events (PTEs), multiple physical illnesses and substance use disorder (SUD) being associated with anxiety-depression, with the older Vietnamese-immigrants reporting greater exposure to these factors. The interaction between key risk factors differed fundamentally when comparing Vietnamese-immigrant and Australian-born samples. Age emerged as the major discriminator, with young Vietnamese-immigrants exhibiting particularly low rates of anxiety-depression.

**Conclusions:**

The findings reported here suggest that core risk factors for anxiety-depression may be universal, but their patterning and interaction may differ according to country-of-origin. The study also highlights the importance of including both standard international and culturally-specific measures to index cross-cultural manifestations of common mental disorders.

## Background

Recent national epidemiologic studies have demonstrated a wide variation in the prevalence of anxiety-depression disorders across countries [1-3]. Comparing immigrant populations from low and middle income countries (LMIC), to compatriots in their country-of-origin and the host population in their country of resettlement (typically high income countries; HIC), offers the potential to develop a greater understanding of the factors contributing to these differences in prevalence rates. Past studies in this field have been undertaken almost exclusively in North America, the focus being on Mexican and Puerto Rican (LMIC) immigrants to the United States [4-6]. Immigrant populations have tended to display lower rates of anxiety-depression relative to the host society, and higher [4,6] or comparable [5] rates to compatriots remaining in the source country. In the only study undertaken outside North America, the rate of anxiety-depression amongst Vietnamese immigrants was also found to be substantially lower than that of the host Australian-born population but higher than a Vietnamese source country sample [7]. The present analysis will extend the findings of our studies amongst Vietnamese populations to investigate for the first time the nature and possible interactions of risk factors in determining the variation in inter-population prevalence rates.

Certain demographic and psychosocial risk factors for anxiety-depression appear to be universal, in particular, female gender [8]; unemployment [9]; lower levels of education [10]; young (versus older) adulthood [11]; comorbid substance use disorder (SUD) [12,13]; physical ill-health [14]; exposure to potentially traumatic events (PTEs) [15]; and in relevant samples, exposure to refugee-specific experiences such as political persecution and forced displacement [15,16]. An important question addressed in the present study is how these universal risk factors contribute to variations in inter-country anxiety-depression prevalence rates. First, we will examine the extent to which inter-country anxiety-depression prevalence differences are associated with variation in the quantity of exposure to risks such as unemployment, poor general health and high levels of trauma exposure [17]. We will then further investigate whether there are ethno- specific risk factor patterns operating as a function of country-group [18]. For example age has been found to operate differently between Hispanic vs. non-Hispanic groups in the United States, with older age being associated with mental disorder in the former group and younger age in the latter [19]. If different risk factor patterns can be established when comparing an immigrant group to their host and source country populations, it will point towards the need to consider possible ecological, cultural and biological differences that may account for the variation in anxiety-depression between populations born in different countries [2].

Ensuring the accurate measurement of anxiety-depression across cultures remains a ‘grand challenge’ in global mental health [20]. There is growing recognition that standard diagnostic measures may be insensitive to culture-specific modes of experiencing and reporting psychiatric symptoms [7,21]. One strategy to address this challenge is to supplement standard diagnostic measures with indigenously-derived measures of anxiety-depression, an approach implemented in the present study. The disadvantage of adding a measure to the assessment of the source country and immigrant group (but not the host population) is outweighed by the risk of under-estimating the prevalence of disorders if only Western-derived measures are used [22].

The present analysis draws on three community mental health surveys administered amongst: 1) Vietnamese refugee-immigrants settled in New South Wales, Australia; 2) a Vietnamese source country sample residing in the Mekong-Delta region of Cần Thơ City and Hậu Giang Province; and 3) the general Australian-born host population [7]. The aim of the study was to compare the Vietnamese-immigrant group with the source and host samples in order to test whether (a) quantitative differences in risk factor profiles accounted for variation in anxiety-depression prevalence between the population groups; or (b) whether there are distinct interactions in risk factors based on country-of-origin or current residency.

## Methods

### Sampling and procedure

#### Vietnamese-immigrant survey

A multistage probability proportional to size (PPS) sampling methodology was applied to 44 census collection districts within 5 local government areas in New South Wales, Australia, resident to 75% of the state’s Vietnamese population. All non-Vietnamese names in phonebook listings were eliminated prior to approaching remaining households (n = 6, 264), initially by letter, followed by a visit from a Vietnamese-speaking interviewer. Within the 1, 413 dwellings identified as housing Vietnamese residents born in Vietnam, one adult respondent (aged over 18 years) was randomly invited for interview. Interviews were undertaken between June 1999 and May 2000, with the majority of interviews conducted in Vietnamese (98%). The study was approved by the Human Research Ethics Committees at the University of Adelaide and University of New South Wales, Australia. The final sample comprised 1, 161 Vietnamese adults, representing an 82.6% response rate, and their average period of resettlement in Australia was 11 years.

#### Mekong Delta Vietnamese survey

The survey was conducted between November 2004 and March 2005 in Cần Thơ City and Hậu Giang Province in the Mekong Delta region of Vietnam, comprising a mixed rural/urban population of approximately 2 million people. The survey applied a multistage probability cluster design. Population PPS sampling was applied to 31 hamlets (16 from 501 hamlets in Cần Thơ and 15 from 487 hamlets in Hậu Giang). Within each hamlet, a random commencement point was used to select 100 consecutive households, with a single adult respondent chosen for interview on the basis of age ranking using a randomized Kish grid (without replacements). The study was approved by the Human Research Ethics Committee at the University of New South Wales, Australia, and reviewed and approved by the Cần Thơ Health Service. The final sample comprised 3, 039 respondents from 3, 100 households (98% response rate). All interviews were conducted in Vietnamese.

#### Australian-born survey

A national survey of mental health was conducted by the Australian Bureau of Statistics (ABS) in 1997, in which 10, 461 consenting adults (aged over 18 years) were interviewed in an Australian-wide stratified multistage probability sample of 13, 624 private dwellings (78% response rate). The present study is based on the interviews of the 7, 961 respondents born in Australia.

### Diagnostic Survey Instruments

In all three surveys, respondents were administered the mood, anxiety and SUD modules of the Composite International Diagnostic Interview (CIDI), version 2.0. The CIDI has been implemented globally [23] and is a lay-administered structured diagnostic interview assessing DSM-IV disorders [24,25]. The index used for the present study was the 12-month diagnosis of either a mood disorder (depression, dysthymia, mania, hypomania or bipolar disorder) and/or an anxiety disorder (generalized anxiety disorder, panic disorder, social phobia, agoraphobia, obsessive-compulsive disorder or posttraumatic stress disorder). The 12-month presence of a SUD, including alcohol/substance harmful use or dependence, was treated as a risk factor for anxiety-depression in the regression analyses (see below).

Both Vietnamese samples completed the 12-month version of the Phan Vietnamese Psychiatric Scale (PVPS) [26]. The measure was developed by gathering indigenously derived expressions of psychological distress amongst Vietnamese populations and then subjecting the item pool to a series of psychometric analyses that yielded three sub-scales: a 26-item depression scale, a 13-item anxiety scale and a 14-item somatization scale [26,27]. Prior testing of the PVPS has demonstrated the instrument’s reliability and validity: test-retest reliability was r = 0.89 for the depression sub-scale, and r = 0.81 for the anxiety sub-scale; internal consistency ranged from 0.93-0.95 for depression and 0.91-0.93 for anxiety [26]. The sub-scale cut-off thresholds were validated at: depression ≥ 1.85; anxiety ≥ 1.45. A previous study has demonstrated sound diagnostic concordance between the CIDI and PVPS for the Vietnamese-immigrant sample used in the present study (depression subscale: area under the curve (AUC) = 0.73, 95% confidence interval (CI): 0.64-0.81; anxiety subscale: AUC = 0.71 (95% CI: 0.64-0.79) [7]. A lower level of diagnostic concordance was demonstrated for the Mekong Delta Vietnamese sample (depression subscale: AUC = 0.65 [95% CI: 0.56-0.73]; anxiety subscale: AUC = 0.64 [95% CI: 0.59-0.7]. In both groups, the PVPS identified the majority of CIDI cases (particularly in the Vietnamese-immigrant sample) but also detected a substantial number of additional cases [7]. Further, the PVPS identified a similar prevalence rate of anxiety-depression across the two Vietnamese samples, whereas the CIDI detected substantially more cases amongst the immigrant than the source country group [7]. PVPS cases also demonstrated similar levels of disability as CIDI-detected cases in both Vietnamese groups, suggesting that the PVPS did not identify persons with a milder level of disturbance [7]. These observations indicate that the CIDI may lack sensitivity in identifying the full range of cases of anxiety-depression amongst Vietnamese populations, particularly in those living in the home country. Hence, to ensure comprehensive coverage of cases of anxiety-depression, we included both CIDI and PVPS cases in examining risk factor profiles amongst the two Vietnamese samples.

PTE exposure was measured by the Traumatic Events Screen (CIDI 2.0 PTSD module), with 14 additional items included from the Harvard Trauma Questionnaire [28] in the Vietnamese surveys due to the particular relevance of these items to the trauma history of Southeast Asian refugees. The HTQ trauma events were mapped onto the CIDI trauma categories to allow for quantitative comparisons between Australian and Vietnamese samples [29].

A common list of medical conditions was included in all surveys covering a range of 12 serious physical health states, including circulatory and endocrine diseases to index general physical health [30].

### Translation

The CIDI was translated into Vietnamese using common translation and blind back-translation methods [31]. Minor language modifications were made to account for small idiom differences between Vietnamese-immigrants and their compatriots living in the Mekong Delta [7]. All three surveys were administered by trained interviewers using prompt cards and on-site computer-assisted personalized interview (CAPI) technology.

### Statistical analyses

#### Data preparation

Statistical analyses were conducted using SAS version 9.1.3 (SAS Institute Inc., Cary, NC, USA). The three samples were weighted according to the age and sex distribution of their respective base populations using national census data. This was conducted to reflect the population socio-demographic characteristics at the time of the survey, taking into account the probability of being sampled and the differential response across the population.

Each sample was stratified according to three age bands: young adults (18–29 years); mid-age adults (30–44 years) and older adults (45+ years), broadly reflective of the probability of being exposed to trauma during the war in Vietnam: the young group would not have been directly exposed; the mid-age group may have been exposed as children; and the older group would have lived through the war as adults.

Other demographic factors examined were: employment (2 categories: “Paid employment/home duties” as the reference group, inclusive of paid employment, active home duties and being a pensioner/too sick to work; and “Unemployed” inclusive of those unemployed and actively seeking paid employment); education (3 categories; “not completed high school”, “completed high school” and “university or vocational training” the later serving as the reference group). We derived three levels of exposure to PTEs: 0 PTEs (no exposure as the reference group); 1–2 PTE types (low exposure); 3 or more PTE types (high exposure) [29]. Medical condition responses were analyzed to provide a composite account of the number of illnesses reported: none (reference group); 1 medical condition; 2 or more medical conditions. Substance use disorder (SUD) was indexed according to two categories (diagnosis present or absent, the later serving as the reference group).

#### Data analysis

A diagnosis for either mood or anxiety disorder was derived from the CIDI for the Australian-born population, and from the combined CIDI and/or PVPS for both Vietnamese populations. The PVPS sub-scale thresholds used were: depression ≥ 1.85; anxiety ≥ 1.45.

Chi-square analyses were first conducted to examine differences between population groups within each demographic factor (p < .05).

Two multivariable logistic regression analyses were then conducted to examine the association between risk factors and anxiety-depression diagnoses: the first between the Vietnamese-immigrant and the Mekong Delta Vietnamese samples, and the second, comparing the Vietnamese-immigrant group with the Australian-born sample. The purpose of these analyses was to examine whether quantitative differences in risk factors accounted for variations in prevalence rates across samples or whether country-of-origin influenced the pattern of risk factors and their interaction. Common risk factors active in all three population groups were included in the model, specifically age, sex, employment status, PTE exposure, medical conditions and SUD. Predictors were tested in three ways: as main effect factors, as 2-way interactions by population group; and higher-order 3-way interactions with population group and age. Reference groups were held constant for both analyses with the exception of age in order to ensure odd ratios fell within the +1 range for age-related interactions between population groups.

Missing values constituted less than 2% of the data in Analysis 1 (Vietnamese-immigrant/Mekong Delta Vietnamese samples), and these participants were excluded from the model. There were no missing values in Analysis 2 (Vietnamese-immigrant/Australian-born samples). We commenced with the full model in which all risk factors and interaction terms were examined. Non-significant variables (p > 0.05) were then iteratively removed in order to refine the final model. Odds ratios (OR) with 95% confidence intervals were used to interpret significant main effects if there were no statistically significant interaction terms. Significant interaction effects were interpreted by reference to prevalence estimates and post-hoc chi-square analyses. The c-statistic was used as a measure of the accuracy of prediction in the final models with values between 0.7 and 0.8 indicating a good fit to the data [32].

## Results

### Demographic characteristics

The socio-demographic characteristics of the three populations are presented in Table 1. The gender distribution was similar across groups (χ^2^ (2) = 1.9, p < .38). The Vietnamese samples were significantly younger (χ^2^ (4) = 334.2, p < .0001), had a lower level of tertiary or equivalent education (χ^2^ (4) = 239.5, p < .0001) and a higher rate of unemployment (χ^2^ (2) = 117.9, p < .0001) than the Australian-born group. The 12-month prevalence of anxiety-depression based on the combined index of CIDI and PVPS cases was 7.0% (95% CI: 5.5% - 8.5%) for the Vietnamese-immigrant group and 4.8% (95% CI: 4.0% - 5.6%) for the Mekong Delta Vietnamese. The rate of anxiety-depression amongst the Australian-born was the highest at 10.2% (95% CI: 9.5% – 10.9%) based on CIDI-only diagnoses.

**Table 1:** Sociodemographic characteristics of population groups

		**Vietnamese-immigrants**			**Mekong Delta Vietnamese**			**Australian-born**			
		**Number**	**%**	**% estimated prevalence of anxiety-depression**	**Number**	**%**	**% estimated prevalence of anxiety-depression**	**Number**	**%**	**% estimated prevalence of anxiety-depression**	**Chi-square group differences**
**Overall**											
	Total for population group	1161	100%	7.0%	3039	100%	4.8%	7961	100.0%	10.2%	
**Gender**											
	Male	572	49.3%	5.9%	1431	47.1%	3.5%	3839	48.2%	7.8%	χ^2^ (2) = 1.9, p<.38
	Female	589	50.7%	8.1%	1608	52.9%	6.1%	4122	51.8%	12.4%	
**Age**											
	18-29 year	371	26.9%	2.0%	1203	39.6%	3.1%	2152	27.0%	11.7%	χ^2^ (4) = 334.3, p<.0001
	30-44 years	493	42.4%	8.6%	1020	33.5%	3.9%	2469	31.0%	12.7%	
	45+ years	297	25.6%	10.4%	816	26.9%	8.5%	3340	42.0%	7.4%	
**Marital Status**											
	Married	741	63.8%	6.7%	2153	70.8%	5.3%	5038	63.3%	8.3%	χ^2^ (2) = 56.7, p<.0001
	Not married	420	36.2%	7.2%	886	29.1%	3.6%	2923	36.7%	13.4%	
**Education**											
	Not completed high school	689	59.4%	7.5%	1937	63.8%	5.1%	4668	58.6%	10.9%	χ^2^ (4) = 239.5, p<.0001
	Completed high school	217	18.7%	5.0%	675	22.2%	5.6%	1179	14.8%	9.8%	
	University/vocational training	255	22.0%	5.8%	426	14.0%	2.3%	2113	26.5%	8.8%	
**Employment**											
	Paid employment/ home duties	1070	92.1%	6.7%	2763	90.9%	4.4%	7638	95.9%	9.7%	χ^2^ (2) = 117.9, p<.0001
	Unemployed	91	7.9%	10.4%	276	9.1%	8.3%	323	4.1%	20.8%	
**PTE exposure**											
	No PTE	492	42.4%	3.9%	2568	84.5%	3.9%	3457	43.4%	6.8%	χ^2^ (4) = 1666.5, p<.0001
	1-2 PTEs reported	401	34.6%	6.3%	404	13.3%	8.8%	3248	40.8%	10.0%	
	3 or more PTEs reported	267	23.0%	13.6%	67	2.2%	18.5%	1257	15.8%	20.1%	
**Substance Use Disorder**											
	Negative diagnosis	1142	98.4%	6.8%	3004	98.9%	4.8%	7262	91.2%	8.6%	χ^2^ (2) = 271.7, p<.0001
	Positive diagnosis	19	1.6%	17.2%	34	1.1%	12.6%	699	8.8%	26.3%	
**Number of self-reported medical conditions**											
	None	851	73.3%	4.5%	2161	71.1%	3.1%	4860	61.0%	9.0%	χ^2^ (4) = 239.5, p<.0001
	1 condition	214	18.4%	10.9%	697	22.9%	7.1%	1886	23.7%	10.9%	
	2 or more conditions	96	8.3%	20.3%	181	5.9%	17.0%	1215	15.3%	13.7%	

Number and % within each demographic variable and weighted prevalence estimates of anxiety-depression (%) for the Vietnamese-immigrant, Mekong Delta Vietnamese and Australian-born populations. Demographic differences between population groups are indicated for each variable category by Chi-square analyses in the right column.

### Multivariable logistic regression analyses

The two multivariable logistic regression models presented in Table 2 yielded c-statistics indicating a moderate to good level of prediction (Vietnamese-immigrant/Mekong Delta Vietnamese analysis 1: c = 0.723; Vietnamese-immigrant/Australian-born analysis 2: c = 0.743). Nonsignificant interaction terms (population group by sex, employment and medical disorder respectively) were excluded from the final modeling.

**Table 2:** The final reduced multivariable logistic regression models

			**1. Vietnamese-immigrant/Mekong Delta Vietnamese Final Model**					**2. Vietnamese-immigrants/Australian-born Final Model**				
**Predictor variables**			**df**	**Wald Chi-Square**	**p value**	**OR**	**95% C.I.**	**df**	**Wald Chi-Square**	**p value**	**OR**	**95% C.I.**
**Main effects**												
	Female (Reference: Male)		1	20.12	<.000	2.0	1.5–2.7	1	109.40	<.000	2.3	1.9–2.7
	Unemployment (Reference: Paid Employment)		1	15.19	<.000	2.3	1.5–3.5	1	19.10	<.000	1.8	1.4–2.4
	Substance Use Disorder (Reference: No SUD)		1	9.66	<.000	4.0	1.7–9.6	1	134.01	<.000	*3.5*	*1.8*–*6.8*
	PTE (Reference: 0 PTE)											
		1–2 PTEs	1	0.00	0.959	1.0	0.8–1.3	1	5.42	0.020	*0.9*	*0.2*–*3.0*
		3+ PTEs	1	19.58	<.000	2.0	1.7–2.3	1	137.12	<.000	*1.9*	*1.6*–*2.3*
	Age group											
		Age: 18–29 years	1	4.64	0.031	*0.6*	*0.4*–*1.0*	Reference			1.0	
		Age: 30–44 years	Reference			1.0		1	15.06	<.000	*1.4*	*1.2*–*1.7*
		Age: 45+ years	1	0.21	0.650	*0.9*	*0.6*–*1.3*	1	0.27	0.601	*0.9*	*0.8*–*1.2*
	Number of medical conditions (Reference: 0 medical conditions)											
		Medical conditions: 1 condition	1	0.16	0.686	1.9	1.3–2.6	1	0.58	0.445	1.0	0.7–1.2
		Medical conditions: 2 or more conditions	1	30.54	<.000	3.9	2.6–5.9	1	36.81	<.000	2.2	1.8–2.7
	Population group (Ref: Vietnamese-immigrant)			5.65	0.017	*1.3*	*1.0*–*1.6*	1	14.52	<.000	*1.3*	*1.1*–*1.5*
**Interaction effects**												
	Population group by Age		2	8.86	0.012			2	18.90	<.000		
	Population group by Age by PTE exposure		4	7.53	0.110			4	12.29	0.015		
								2	14.99	<.001		
	Population group by Age by Substance use disorder											

Presented are the final reduced multivariable logistic regression models for Analysis 1: Vietnamese-immigrant/Mekong Delta Vietnamese; Analysis 2: Vietnamese-immigrant/Australian-born. Both main and interaction effects are included, with reference group indicated in the left column where applicable. Interpreting findings at the main effect level must be treated with caution in the presence of a significant interaction effect (indicated by italicized OR and 95% C.I. indices). Note: different reference groups are used for the age variable between analysis 1 and 2.

### Main effect risk factors common to all three populations

Being female and being unemployed emerged as risk factors common to all populations in both models, with each of these individual factors doubling the odds for having an anxiety-depression diagnosis. The presence of more than one medical condition was associated with a four-fold increase in odds in the Vietnamese–immigrant/Mekong Delta model, and a two-fold increase in the Vietnamese-immigrant/Australian-born model (Table 2).

The highest category of PTE exposure (3 or more PTE categories) was a significant risk factor as a main effect in the analysis involving the Vietnamese samples (p < .0001; analysis 1), doubling the odds for anxiety-depression. There was a substantial difference between the Vietnamese samples in the proportion falling within the high PTE exposure category: 23% of the Vietnamese-immigrant group compared to 2.2% of the Mekong Delta sample (χ^2^ (1) = 496.2, p < .000). PTE exposure was also associated with increased risk for anxiety-depression in analysis 2, but significantly interacted with population and age (see below). The prevalence of SUDs was low amongst both Vietnamese groups relative to the Australian-born sample (Vietnamese-immigrants: 1.6%; Mekong Delta Vietnamese: 1.1%; Australian-born: 8.8%). Nevertheless, a comorbid SUD diagnosis emerged as a significant risk factor as a main effect for the Vietnamese in both settings (analysis 1), increasing the odds by a factor of 4 for anxiety-depression.

### Interaction Effects

#### Population by age

A significant interaction between population group and age was evident in both regression analyses (Table 2) but the pattern differed according to the population under consideration (prevalence rates by age are presented in Table 1). The risk for anxiety-depression increased with age in both Vietnamese populations (analysis 1), with particularly high rates of anxiety-depression in the oldest age groups. The pattern of increased prevalence emerged at an earlier age amongst the Vietnamese-immigrants, with the 30–44 year group showing greater risk (8.6%) than their Mekong Delta Vietnamese counterparts in the same age band (3.9%: χ^2^ (1) = 13.7, p < .000).

When comparing immigrants with the host sample (analysis 2), there was a trend for older Vietnamese-immigrants to show a greater prevalence of anxiety-depression (10.4%) than older Australians (7.4%; χ^2^ (1) = 3.58, p = .059). There was a notable reversal of that pattern for both the mid-age band (Vietnamese-immigrants: 8.6%; Australian-born: 12.7%; χ^2^ (1) = 6.58, p = .009), and a pronounced difference for the youngest age group (Vietnamese-immigrants: 2.0%; Australian-born: 11.7%; χ^2^ (1) = 6.47, p = .01).

#### Population by age by PTE exposure

**Figure 1:**
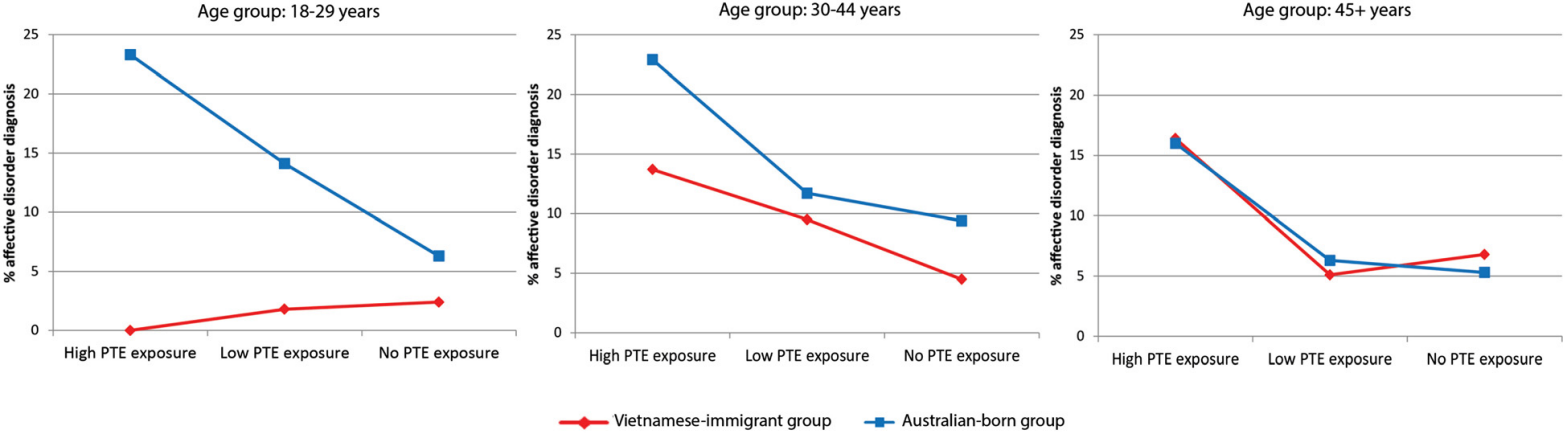
**Population differences in risk posed by PTE exposure and age between Vietnamese-immigrant and Australian-born groups.** Variations in risk posed by PTE exposure in the Vietnamese-immigrant (red) and Australian-born groups (blue) across age group: 18–29 years; 30–44 years; 45+ years. Each figure displays the percentage prevalence estimates of 12-month anxiety-depression on the vertical axes. High PTE load corresponds to exposure to 3 or more PTE types; low PTE load refers to exposure of 1–2 PTE types; no PTE reflects no PTE reported. Prevalence rate data is also presented in Table 3.

In the Vietnamese-immigrant/Australian-born model (analysis 2), a significant 3-way interaction emerged for population group by age by PTE exposure (p = 0.015; Table 2; prevalence rates by age and PTE exposure are displayed in Table 3). High PTE exposure posed a large and similar risk for anxiety-depression for older Australian and older Vietnamese-immigrants (Vietnamese-immigrants: 16.4%; Australian-born: 16%). Nonetheless, a substantially larger proportion of the Vietnamese-immigrants fell into the high PTE class (42.9%, compared to their Australian-born counterparts, 16.1%; χ^2^ (1) = 133.7, p < .000), accounting in part for the overall higher prevalence of anxiety-depression in older Vietnamese. For the 30–44 year age band, anxiety-depression prevalence for Vietnamese-immigrants was half that of the Australian-born group (Vietnamese-immigrants: 13.7%; Australian-born: 22.9%; χ^2^ (1) = 4.2, p = .041), despite a greater portion of Vietnamese-immigrants reporting high PTE exposure (23.0%, compared to the Australian-born sample 17.1%; χ^2^ (1) = 10.2, p < .001). Fewer young Vietnamese-immigrants reported high-PTE exposure (7.1%) compared to the young Australian born group (13.9%; χ^2^ (1) = 13.1, p < .000).The young adult Vietnamese-immigrant sample exhibited no association between high PTE exposure and anxiety-depression, whereas the typical dose–response association held for the young Australian-born cohort (23.3%; see Table 3). These differences by age and PTE exposure across population groups are displayed in Figure 1.

**Table 3:** Presents disaggregated for significant interaction effects in analysis 2

				**Age: 18–29 years**			**Age: 30–44 years**			**Age: 45+ years**		
				**Number**	**% within age sample**	**% prevalence of anxiety-depression**	**Number**	**% within age sample**	**% prevalence of anxiety-depression**	**Number**	**% within age sample**	**% prevalence of anxiety-depression**
**Significant interaction terms analysis 2: Vietnamese-immigrant/Australian-born**												
	**PTE exposure**											
		Vietnamese-immigrant group										
			High PTE exposure	26	7.1%	0.0%	114	23.0%	13.7%	128	42.9%	16.4%
			Low PTE exposure	116	31.1%	1.8%	198	40.1%	9.5%	88	29.6%	5.1%
			No PTE exposure	229	61.8%	2.4%	182	36.8%	4.5%	82	27.5%	6.8%
		Australian-born group										
			High PTE exposure	300	13.9%	23.3%	421	17.1%	22.9%	536	16.1%	16.0%
			Low PTE exposure	828	38.5%	14.1%	1027	41.6%	11.7%	1392	41.7%	6.3%
			No PTE exposure	1025	47.6%	6.3%	1021	41.3%	9.4%	1411	42.3%	5.3%
**Substance use disorder (SUD)**												
		Vietnamese-immigrant group										
			SUD diagnosis	9	2.3%	0.0%	6	1.2%	27.6%	4	1.3%	38.3%
			No SUD diagnosis	362	97.7%	2.1%	487	98.8%	8.4%	293	98.7%	10.0%
		Australian-born group										
			SUD diagnosis	360	16.7%	20.9%	236	9.6%	32.2%	103	3.1%	31.6%
			No SUD diagnosis	1792	83.3%	9.8%	2233	90.4%	10.5%	3237	96.9%	6.7%

Incidence (number and%) and prevalence rates (%) for anxiety-depression disaggregated by age group, PTE exposure and SUD diagnosis in the Vietnamese-immigrant and Australian-born populations (Analysis 2).

#### Population by age by substance use disorder

There was a significant interaction of SUD and age for anxiety-depression when comparing the Vietnamese-immigrant and Australian-born groups (analysis 2: population group by age by SUD interaction: p < .001; Table 2, prevalence rates Table 3). SUD was not associated with anxiety-depression amongst young Vietnamese-immigrants but incurred a considerable risk amongst the young Australian-born (20.9%). In older age groups, similar associations between SUD and odds for anxiety-depression in both samples were observed: for instance, prevalence was 38.8% in the older Vietnamese-immigrant sample and 31.6% in the older Australian-born group.

#### CIDI-only anxiety-depression cases in the Vietnamese samples

To test whether the addition of the PVPS biased the findings for the Vietnamese samples, we repeated the key analysis for age using the CIDI results alone, a procedure that necessitated the pooling of both Vietnamese groups to achieve adequate statistical power. The results confirmed the pattern for the combined CIDI-PVPS findings, namely that younger adult Vietnamese (0.8% in the 18–29 year group) had a lower prevalence rate of anxiety-depression than the older categories (2.9% in the 30–44 year age group; 2.0% in the 45+ year age group).

## Discussion

Our study aimed to examine factors underlying the consistent stepwise anxiety-depression prevalence rate patterns across host country populations (highest rates), resettled immigrant groups (intermediate rates) and source country populations (lowest rates). The findings provide evidence that risk factors vary not only in quantity between populations, but in their interaction based on the country-of-origin of the population. Risk factor profiles were similar between the Vietnamese-immigrant sample and the source Vietnamese population; but there were differences in the interaction of risk factors when comparing the Vietnamese-immigrant and the Australian-born samples. An analogous pattern has been observed for cross-national suicide rates, in which prevalence rates are largely constant between immigrants and their source country populations, but differ from those of the host society [33].

Risk factors that were common to all populations included being female, unemployment, and having poor physical health, an observation that supports the universal importance of these influences. The two Vietnamese populations showed consistency in their risk factor profiles (analysis 1), with the differences being entirely quantitative in nature. Specifically, older Vietnamese-immigrants showed higher rates of PTE exposure, number of medical conditions and SUD diagnoses than their Mekong Delta compatriots — risk factors that in concert exerted a greater burden of anxiety-depression in that group. The pattern for PTE exposure is consistent with the general dose–response relationship with anxiety-depression disorders observed in other studies amongst refugee groups exposed to war and displacement traumas, including those involved in the Southeast Asian conflicts during the 1960-70s [15,16,34,35]. The higher rates of physical illness and SUD amongst older Vietnamese-immigrants may reflect the effects of exposure to war and the stress of forced migration.

In contrast, there was a marked difference in the pattern and interaction of risk factors between the Vietnamese-immigrant and Australian-born samples (Analysis 2). Age emerged as the key discriminator: the older cohort demonstrated higher rates of anxiety-depression in the Vietnamese-immigrant group; whereas the youngest cohort exhibited the greatest risk for anxiety-depression in the Australian-born sample. Older age also posed a greater risk in the Mekong Delta Vietnamese group (analysis 1). This contrast in age risk between both Vietnamese and Australian-born populations is consistent with other data from LMIC and HIC countries [11,36,37], a finding that appears to be independent of the impact of medical disorders with advancing age [36,37]. It is vital therefore to explore further why age exerts such a powerful but variable influence on the prevalence of anxiety-depression across countries [36].

PTE exposure was low and showed no association with anxiety-depression amongst young Vietnamese-immigrant adults, in contrast to the findings for the older Vietnamese-immigrants who were highly exposed to conflict-related trauma [26]. Young Vietnamese-immigrants differed markedly from the young Australian-born cohort who reported higher levels of PTEs and showed greater vulnerability to their impact in relation to risk of anxiety-depression. It is possible that specific factors not measured in our study act to protect younger Vietnamese-immigrants from anxiety-depression, including greater constraints on the activities of young adults, a greater level of social interdependence and high levels of familial support [38,39]. These social factors may protect young adults from encountering traumas in the first instance, and buffer those who are exposed against any deleterious emotional impact. Young Vietnamese-immigrants may also be protected against developing SUDs by virtue of the same cultural protective influences, a possibility that is supported by other studies amongst young Asian immigrant samples [40].

The present study adds important information to the debate regarding the universality or otherwise of the symptoms and determinants of common mental disorders such as anxiety-depression. Proponents of a universalistic position assert that disorders such as anxiety-depression are comparable across cultures, differing only in their surface manifestations [2]. The opposing position holds that the origins and nature of disorders vary fundamentally across cultures and contexts [41-43]. The findings reported here suggest that core risk factors may be universal, but their patterning and interaction may differ fundamentally across cultural and population groups. The findings also offer support to recent commentaries focusing on the diagnostic revisions in preparation for DSM-V, by demonstrating the importance of including culturally sensitive measures in assessing anxiety-depression disorders at a global level [18,21,44]. Although adding an indigenous measure means that assessment protocols are not strictly commensurable across cultures, this strategy offers the advantage of ensuring that prevalence rates are not under-estimated through using only Western-derived measures, particularly amongst populations from East Asia [22,44]. Our indigenous measure showed a moderate level of concordance with the CIDI amongst the Vietnamese-immigrants, but at the same time identified cases not detected by that measure [7]. Additionally, the risk associated with age remained consistent when the analysis was based on only CIDI diagnoses for the combined Vietnamese sample. Overall, the findings point to the value of combining indigenous and international diagnostic measures in transcultural comparative studies of this type.

Limitations of the study need to be acknowledged. First, the Mekong Delta represents just one region in a culturally diverse Vietnam; the site was selected because most Vietnamese-immigrants in Australia originate from the south of Vietnam. There would be benefit in replicating the findings in a nationally representative sample. There was a difference in the timing of the surveys forming the basis of the current analysis, with the Mekong Delta survey being undertaken more recently. We note, however, that if there was a process of secular shift, it would presumably have lessened differences between Vietnamese-immigrants and the Mekong Delta Vietnamese samples because of the recent acceleration of Westernization in Vietnam. Although response rates were high across all three samples, variation in participation may have influenced the proportion in each sample with and without anxiety-depressive disorders.

It was not possible to assess the specific impact of culture and migration experiences since these factors were only relevant to one group, the Vietnamese-immigrants. Clinical recalibration of the CIDI with other DSM–IV-based diagnostic instruments was not undertaken in the surveys included in the current analysis limiting definitive statements about the validity of the CIDI in assessing DSM-IV diagnoses in these populations. Studies undertaken among East Asian populations, including neighboring China, have identified adequate concordance between the CIDI and measures such as the Structured Clinical Interview for DSM–IV in settings despite recording similarly low prevalence estimates [22].

## Conclusions

The findings suggest that country-of-origin may exert a powerful impact on the interaction of common risk factors associated with anxiety-depression, an effect that persisted in the immigrant group even though it had resided in the host society for more than a decade. Further pursuit of this theme may throw important light on the origins and pathogenesis of anxiety-depressive disorders, and their expression and prevalence internationally. Age of risk is of particular importance, an issue worthy of further study given that there is evidence of a diametrically different pattern across populations from LMIC and HICs. In particular, if it is possible to determine why young adults from LMICs have such a low prevalence of anxiety-depression, that knowledge could be translated into improving prevention and intervention strategies for young adults in HICs who are at higher risk of developing these disorders.

## Competing interests

The authors report no competing interests.

## Authors’ contributions

BJL oversaw the analysis and interpretation of the data, and produced the initial and final manuscript. TC conducted the statistical analyses and assisted in interpreting the findings. DS contributed to the design of the Vietnamese-immigrant and Mekong Delta Vietnamese surveys, interpretation of analyses, and to the development of the manuscript. TTBP conducted and managed the Vietnamese surveys reported. MGN contributed to the design and acquisition of the Mekong Delta Vietnamese survey. ZS conceptualized the design and oversaw the implementation of the Vietnamese-immigrant and Mekong Delta Vietnamese surveys, and was substantially involved in the production of the manuscript. All authors have read and approved the final manuscript.

## Pre-publication history

The pre-publication history for this paper can be accessed here:

http://www.biomedcentral.com/1471-244X/13/329/prepub
